# Causes and management of acute oncological pain: a narrative review

**DOI:** 10.1111/anae.16512

**Published:** 2025-01-08

**Authors:** Helen Laycock, Candice Ramdin, Justin Grayer, Matthew R. D. Brown

**Affiliations:** ^1^ Department of Pain Medicine Great Ormond Street Hospital London UK; ^2^ Department of Pain Medicine San Fernando General Hospital, Southwest Regional Health Authority Trinidad and Tobago; ^3^ Adult Psychological Support Service The Royal Marsden Hospital London UK; ^4^ Department of Pain Medicine The Royal Marsden Hospital London UK; ^5^ The Institute of Cancer Research London UK

**Keywords:** acute pain, cancer, mucositis, neuropathy, opioids, radiotherapy

## Abstract

**Introduction:**

Acute pain in cancer is an important but often overlooked feature of many patients' oncological journey. Cancer‐related pain is associated commonly with more persistent pain states caused by both the disease and its treatment, but there are numerous causes of acute pain which can develop in patients with cancer. This pain is frequently severe, can be challenging to manage and its suboptimal control can directly impact on oncological outcomes. This narrative review provides an overview of several causes of acute pain in patients with cancer and management approaches.

**Methods:**

A focused literature review was conducted to encompass the search terms ‘acute pain’, ‘oncology’ and ‘cancer’ in adult and paediatric populations.

**Results:**

Acute pain is common in patients with cancer with a number of pain generators identified. Broadly, these are disease‐ and treatment‐related but commonality in pain mechanisms and features are present. Importantly, these pain states do not occur in isolation; a patient may experience multiple acute pain episodes during their oncology journey.

**Discussion:**

As the oncological treatment landscape shifts and increasing numbers of novel treatments are employed, the number of causes of acute pain in patients with cancer rises. This pain is often managed by non‐pain specialists and suboptimal control has a variety of deleterious effects. It is important that awareness of acute pain in the oncological population is increased and treatment approaches, which adopt a biopsychosocial structure, are optimised.

## Introduction

Acute pain management associated with cancer poses a significant clinical challenge. With an ageing population and increasing levels of lifestyle‐related diseases, cancer diagnoses are rising globally, representing the first or second leading cause of premature death (i.e. at ages 30–69 y) in 134 of 183 countries [1]. The predicted global cancer burden will exceed 27 million new cancer cases per year by 2040, a 50% increase on the estimated 18.1 million cancers in 2018 [1]. Pain is a common symptom and feature of all stages of the disease, from diagnosis through different phases of active treatment, to either survival or advanced disease and death [2]. However, its optimal management continues to prove elusive [3].

Acute pain is defined as “*the physiologic response and experience to noxious stimuli that can become pathologic, is normally sudden in onset, time limited and motivates behaviours to avoid actual or potential tissue injuries*” [4]. Apart from the chronological differences between acute and chronic pain (acute pain resolves within 3–6 months while chronic pain persists beyond this time‐point [5]), acute pain is synonymous with nociceptive pain; pain that arises from actual or threatened damage to non‐neural tissue. Nociceptive pain is triggered by a noxious stimulus (either mechanical, thermal or chemical) that activates peripheral nociceptors and sends impulses along myelinated A δ and unmyelinated C fibres to the spinal cord. These fibres synapse in the spinal cord before ascending to the thalamus, hypothalamus, reticular system and cortex of the brain, where pain is perceived. Chronic pain is predominantly the manifestation of dysfunction and damage to this somatosensory system. This difference is important when considering therapeutic approaches, as the distinct cellular mechanisms between acute and chronic pain result in different pharmaceutical targets.

Managing acute oncological pain is important. Poorly controlled acute pain impacts an individual's physiology by triggering increased sympathetic nervous system activity leading to the release of stress hormones, increased heart rate and tissue oxygen requirements, and immunomodulatory effects [6]. These contribute directly to increased rates of postoperative complications and morbidity with complications affecting myriad organ systems, ranging from ileus through to myocardial infarction [7, 8]. From a health service perspective, inadequately controlled acute pain leads to a longer duration of stay, reductions in health system efficiency and lower patient satisfaction [9, 10]. Acute pain also increases the risk of developing challenging‐to‐treat persistent pain states [11] as well as directly influencing the aberrant use of opioid analgesics [12].

The classical perception of cancer‐related pain focuses on the concept of pain generated by the disease itself, in the form of direct local and loco‐regional effects of a growing tumour. When viewed through this prism, management has often aligned with chronic pain approaches or, in advanced disease, occupied by a unique niche employing neuro‐ablative procedures (i.e. coeliac plexus blocks). This review approaches the challenge of cancer pain management from a different perspective – that of acute pain states encountered in the oncology population. This theme has received much less exposure and consideration yet represents the most frequently encountered situation for many clinicians who work outside specialist oncology centres. We aim to provide an overview of the types of acute pain states encountered, including more unusual causes of pain in patients with cancer. Treatment approaches that adopt and encompass the biopsychosocial model will be discussed.

## Methods

A focused literature review was conducted to encompass the search terms ‘acute pain’, ‘oncology’ and ‘cancer’, including adult and paediatric populations. This strategy was determined to ensure all aspects of patient pathway from diagnosis onwards were considered, as acute pain can occur at all parts of a patient's oncological journey. We excluded articles that focused on chronic pain or end of life care, which are outside the scope of this review.

## Results

### Disease‐related pain

Disease‐related cancer pain is the scenario thought of frequently when ‘cancer pain’ is considered. For solid tumours, the uncontrolled mitotic process leading to their formation also involves the interplay between cancerous cells and the cells of the tumour micro‐environment [13]. The micro‐environment is a complex ecosystem comprising a multitude of cell types including immunocytes; fibroblasts; neurones; and endothelial cells [14]. As a tumour matures, communication between these cells accelerates with ‘crosstalk’, driving important processes supporting the growing tumour such as angio‐ and neurogenesis [15]. Many of the nerves growing into the tumour micro‐environment are sensory. They are hypersensitised by immune‐ or cancer‐derived signals, such as cytokines, growth factors and release neurotransmitters such as substance P and calcitonin gene‐related peptide. In turn, these influence tumour growth by interacting with the immune system and contributing to inflammatory processes [14, 15, 16, 17]. The anatomical location of a tumour influences pain experienced as those closely located to neuronal structures will compress and irritate them. Cancers characteristically associated with severe intractable pain are intimately associated with nerve plexuses (e.g. pancreatic cancer, head and neck cancer and cervical cancer). Perineural invasion, the process in which cancer cells infiltrate in or around nerves, can also cause severe pain alongside facilitating cancer spread [18].

Paraneoplastic syndromes are a diverse group of disorders that occur in patients with malignancies but are not directly attributable to the local presence of cancer cells, metastatic spread or the impact of cancer treatment. These syndromes arise from indirect effects of cancer, primarily mediated by an immune system response to tumour antigens or by the secretion of functional peptides or hormones by the tumour itself [19]. Antibodies or immune cells target not only the tumour but also the peripheral nervous system, leading to the development of peripheral neuropathies, often manifesting as acute, severe pain.

Paraneoplastic neuropathies are associated most commonly with small‐cell lung carcinoma, lymphoma and ovarian cancer. They are often linked to the presence of onconeural antibodies (e.g. anti‐Hu and anti‐CV2/CRMP5) or neuronal surface antibodies (CASPR2 and LGI1) [20] that target neuronal structures, leading to inflammation, demyelination or axonal degeneration; this results in neuronal dysfunction, sensory disturbances and pain [21]. The pain can be widespread or focal, often affecting the limbs and extremities, and may be accompanied by other neurological deficits such as motor weakness or autonomic dysfunction. Management typically involves treating the underlying malignancy and using immunosuppressive therapies, but pain control can present a major challenge due to the often‐progressive nature of the neuropathy.

### Treatment‐related pain: radiotherapy

Radiotherapy, a component of many oncology treatment protocols, may lead to acute pain. The exposure of cells to ionising radiation leads to cellular DNA changes, resulting in cell death. The principle of radiotherapy treatment is that healthy cells have a superior capacity for DNA to repair following sub‐lethal damage from radiation when compared with cancerous cells [22]. The development of radiotherapy‐related toxicity is influenced by a number of factors that include radiation dose; tissue volume; and the underlying vulnerability of tissues to radiation. However, normal non‐cancerous cells can be damaged. Cellular damage caused by radiotherapy can result in either early or late (≥ 90 days from radiotherapy) [23] toxicities.

Early toxicities are important for acute pain. These involve cell loss from tissues possessing a high cellular turnover, such as mucosa or epidermis. Mucositis, cutaneous desquamation and tumour ‘pain flare’ are the acutely painful phenomena encountered most commonly following radiotherapy [24, 25]. Radiation‐induced oral mucositis results from acute inflammation of the mucosa of the mouth, tongue and pharynx [26], with a typical course encompassing four stages: an initial inflammatory phase; an epithelial phase; an ulcerative phase; and a healing phase [27]. The duration of this process can last anywhere from a week to several months [28]. It is not only intensely painful but can be a significant barrier to adequate nutrition and hydration, and may interrupt or limit the intended radiotherapy treatment. This represents a major challenge, especially in head and neck cancers [29]. Radiation‐induced skin reactions are common, occurring in approximately 95% of patients having radiotherapy [30]. The severity varies from localised erythema through to moist desquamation, ulcers and skin necrosis. Dermatological toxicity is a particular issue in patients receiving treatment for anorectal tumours and some gynaecological cancers. Severe pain can make positioning for each fraction of radiotherapy unpleasant and, in extreme cases, can jeopardise completing the course of treatment.

Pain flare is an adverse effect of radiotherapy for bone metastases that occurs in approximately 40% of patients [31]. It can be defined in one of two ways: as an a priori 2‐point increase in worst pain score using a 0–10 numerical rating scale when compared with baseline, with no decrease in analgesic intake; or as a 25% increase in analgesic intake with no decrease in worst pain score. To distinguish from a general progression of pain, analgesic intake and pain scores need to return to baseline after the flare [32]. This phenomenon is often observed within a few days of treatment and can last up to a week before subsiding. The mechanism underlying pain flare is multifactorial and is thought to include localised inflammation caused by damage to tissues, leading to the release of cytokines such as tumour necrosis factor‐α (TNF‐α); interleukin‐1 (IL‐1) and prostaglandins; stimulation of the highly innervated periosteum; increased activation of osteoclasts; and tumour necrosis [33, 34]. Due to the temporary nature, if the initial flare can be managed the patient is likely to experience good resolution of their pain longer term. The risk of experiencing pain flare is included in patient information and informed consent for radiotherapy treatment. While pain flares may be managed with increased doses of analgesia, this approach may be associated with worsening of adverse effects. Patients generally favour a preventative approach and dexamethasone can be administered as an effective prophylactic drug at the time of radiotherapy [35].

### Treatment‐related pain: chemotherapy

Systemic anticancer treatment, namely chemotherapy, is classically associated with chemotherapy induced peripheral neuropathy, a chronic, cumulative sensory neuropathy which develops over the course of chemotherapy [23]. Some chemotherapy drugs can be associated with more acute pain. Oxaliplatin, a platinum analogue used widely in gastrointestinal cancers, is associated with an unpleasant acute cold‐sensitive neuropathy affecting the hands, feet and oropharynx in 80–90% of patients [36]. This can arise within hours of receiving oxaliplatin and may last up to 72 h. The underlying mechanism is thought to relate to oxaliplatin modulating the transcription of distinct ion channels that shape sensory neuronal cold responses [37]. The presence of this phenomenon is a marker for the development of more severe longer‐term chemotherapy‐induced peripheral neuropathy [38].

Due to the rapid turnover of the cells of the nail matrix, toe and fingernails are particularly vulnerable to damage from chemotherapy. Certain nail toxicities may be acutely painful. Onycholysis, the separation of the nail plate from the nail bed, is associated with taxane‐based chemotherapy and, when haemorrhagic, can cause severe pain [39]. Other painful nail toxicities include subungual haematomas and paronychia [39]. Specialist dermatological input to assist with the management of patients presenting with painful nail toxicity is advised.

Some cutaneous toxicities due to chemotherapy may also be painful. Palmar‐plantar erythrodysaesthesia or ‘hand‐foot syndrome’ can occur in patients receiving 5‐fluorouracil, capecitabine, sunitinib, doxorubicin or epirubicin, and manifests as skin blistering, ulceration, erythema and burning pain [40]. This can be particularly troublesome in patients who are older and frail in whom the pain can impact mobility and functional levels significantly, necessitating changes in oncology treatment [41]. The presence of associated dysaesthesia (unpleasant, abnormal sensations) can lead to diagnostic confusion and clinicians who have been referred to patients with painful chemotherapy‐associated peripheral neuropathy accompanied by cutaneous changes should consider palmar‐plantar erythrodysaesthesia.

Chemotherapy‐related mucositis is a complex, multifactorial process involving damage to the mucosal lining of the gastrointestinal tract, particularly the oral cavity. The cytotoxic effects of chemotherapy damage the DNA of basal epithelial cells directly and this results in the production of reactive oxygen species. These reactive oxygen species, alongside pro‐inflammatory cytokines, amplify tissue injury. Ulceration represents the most painful and clinically significant phase of mucositis, as it exposes underlying tissues and nerves [42]. Acute pain associated with mucositis can severely impact a patient's ability to speak, swallow saliva, eat, drink and maintain adequate nutrition, thereby diminishing their overall quality of life and potentially necessitating modifications to their cancer therapy regimen [43]. Effective pain management in mucositis has been highlighted as a key element of care and is crucial, not only for patient comfort, but also to avoid complications such as acute kidney injury due to dehydration and ensuring the continuity and efficacy of cancer treatment [41].

Immune checkpoint inhibitors work by stimulating the immune system's ability to attack cancer but can also trigger immune‐mediated damage in healthy tissues, such as nerves [44]. Neurological immune‐related adverse events caused by immune checkpoint inhibitors often present with pain, affecting the central and peripheral nervous systems [45]. This can manifest as neuropathic pain, such as severe burning; tingling; or shooting pains. Other conditions, such as myasthenia gravis or aseptic meningitis, may present with muscle weakness and headaches, contributing to discomfort and pain. Pain management involves discontinuation of immune checkpoint inhibitors and initiation of immunosuppressive treatments such as corticosteroids; intravenous immunoglobulin; or plasmapheresis (depending on the severity of the condition). The challenge with treating immune checkpoint inhibitor‐induced pain is similar to that encountered with graft‐vs.‐host disease, as the pain may persist even after the neurological symptoms improve, sometimes leading to chronic pain syndromes. Early identification and multidisciplinary approaches are crucial to providing effective pain control.

### Treatment‐related pain: procedural

Surgical‐ and procedure‐related acute pain are important, as pain following surgery is a combination of nociceptive, acute neuropathic and inflammatory elements that serves a distinct biological purpose, namely optimising conditions for wound healing. Numerous and complex cellular processes are triggered when injury to tissues occurs. The skin is densely populated by a large number of neurone terminal fibres, cutaneous cells and immunocytes, and following the noxious insult of surgery, these cells release a slew of pro‐inflammatory signalling molecules into both the local and loco‐regional environment‐stimulating nociceptors [46].

A number of non‐surgical ‘minimally invasive’ procedures that patients undergo may also be associated with significant acute pain. Percutaneous interventional radiology techniques are used commonly to ablate isolated metastases in various organs including the liver and lungs. Pain following radiofrequency ablation of liver metastases is a common complication, often presenting as acute localised discomfort in the upper right quadrant or referred pain in the shoulder due to diaphragmatic irritation. This pain typically peaks within 24–48 h post‐procedure and gradually subsides within a week [47]. The intensity of pain correlates with the size and number of ablation zones, as well as proximity to sensitive structures like the liver capsule. Mechanistically, it is thought to arise from thermal injury to surrounding tissues, including the liver parenchyma and adjacent nerves.

Graft‐vs.‐host disease is a condition that occurs when immune cells, particularly T‐cells, from a donor (the graft) attack the recipient's (the host's) tissues and organs. This is most common after an allogeneic haematopoietic stem cell transplantation. Graft‐vs.‐host disease primarily affects the skin, gastrointestinal tract and liver [48]. Acute pain is a frequent and challenging complication of the condition. In the skin, acute graft‐vs.‐host disease manifests as a painful erythematous rash or severe blistering. Gastrointestinal involvement, often characterised by mucosal ulcerations, can lead to intense abdominal pain, cramping and diarrhoea, while hepatic graft‐vs.‐host disease result in right upper quadrant pain due to liver inflammation [49]. The immune‐mediated attack on host tissues involves donor T‐cells recognising recipient antigens, leading to a release of pro‐inflammatory cytokines, such as TNF‐α and IL‐6, which further exacerbate tissue damage and pain [50].

Granulocyte colony‐stimulating factor (G‐CSF) is used commonly to stimulate the production of neutrophils, particularly in patients undergoing chemotherapy to reduce the risk of febrile neutropenia. However, a well‐recognised adverse effect of G‐CSF is acute bone pain, affecting approximately 20–30% of patients [51]. This pain is thought to result from increased activity in the bone marrow micro‐environment, causing bone marrow expansion, as well as stimulation and sensitisation of sensory nerves due to heightened cytokine production and inflammation. Pain manifests typically in areas of high trabecular bone content such as the femur, pelvis and sternum. It is transient but can significantly impact quality of life during treatment. Managing G‐CSF‐induced bone pain can be challenging as it may be refractory to conventional analgesics. Nonsteroidal anti‐inflammatory drugs are typically the first line of treatment [52]; however, they may be insufficient for some patients. Loratadine, a non‐sedating antihistamine, has emerged as a possible alternative, with some studies suggesting its effectiveness in reducing G‐CSF‐induced pain, potentially by inhibiting histamine‐mediated pathways involved in inflammation [53].

### Acute oncological pain in children

Children display different cancer disease patterns to adults, with leukaemia, brain and spinal cancer and lymphoma the most common cancers in children, compared with breast, prostate, bowel and lung in adults. The incidence of childhood cancer has remained relatively stable (around 18.6/100,000 over the last 50 years [54]); however, 5‐year survival rates have increased due to advanced treatment options that span extensive surgery, radiotherapy and chemotherapy [55]. These therapies come with significant medical consequences, including severe pain.

Many causes of oncological pain overlap with those highlighted for adults, but those associated with treatment are most common [56]. Children undergo multiple painful procedures during the treatment journey that include accessing venous ports, regular bone marrow aspirates and lumbar punctures for diagnosis or to deliver intrathecal chemotherapy. While sedation or general anaesthesia is often used, acute pain extends beyond this period. A mean incidence of 50% of children receiving chemotherapy experience mucositis‐associated pain, one of the most debilitating pains children with cancer experience [57]. Acute pain management is important to facilitate mouth care that removes secondary debris, reducing the risk of secondary infection, as it can be intolerable in children and often requires parenteral opioids and adjuncts.

Veno‐occlusive disease (also known as sinusoidal obstruction syndrome) is a potentially life‐threatening condition most often associated with haematopoietic stem cell transplantation. This has a higher incidence in children compared with adults. Damage to liver sinusoidal epithelium from conditioning chemotherapy regimens, cytokine or endotoxin release, leads to extravascular deposition of cellular debris, downstream embolisation and occlusion of microcirculation. This causes hepatic dysfunction, ascites and associated pain from distension of the abdomen or hepatic capsule [58].

Neuropathic pain can occur following surgery but is mostly experienced secondary to chemotherapy drugs (particularly vincristine). This can be treatment‐limiting and presents as either acute neuropathic pain or pain from chemotherapy‐induced peripheral neuropathy [59]. More recently monoclonal antibody treatments have gained limited licence for use in children, e.g. in high risk neuroblastoma disease, the most common extracranial paediatric solid tumour [54]. Monoclonal antibody therapy can cause neuropathic pain that may limit treatment, as the monoclonal antibody directed against the specific antigen found on neuroblasts (disialoganglioside (GD2)) also binds to GD2 positive peripheral nerves [60].

### Management approaches to pain

The use of a biopsychosocial framework to structure the assessment and management of acute cancer pain is important, yet we propose the addition of ‘onco’ to this framework. A comprehensive understanding of the patient's current oncological status, future plans (both treatment and investigations) coupled with excellent communication with their oncologist is imperative. This ensures co‐ordinated working occurs, especially important when potential pain therapies require a risk:benefit analysis that needs to include the stage of a patient's cancer and their quality of life. As with all pain management, accurate pain assessment is key to guiding treatment and monitoring response. Unidimensional pain scores are often integrated into observation early warning score charts, with the inclusion of triggering scores to prompt intervention. Use of pain descriptors can be helpful to differentiate between types of pain and help guide management.

Analgesic drugs are key to acute cancer pain management. A combination of drugs is often deployed in a multimodal analgesic plan. The selection of such a regimen is not a case of one size fits all; rather regimens should be tailored to individual patients, with consideration of risk factors; patient preferences; and the nature of the pain being treated. The European Society for Medical Oncology recommends adopting an integrative approach that includes primary antitumour treatments, interventional analgesic therapy and a variety of non‐invasive techniques [61]. A stepwise approach to medication choice has long been advocated, dating back to the World Health Organization (WHO) analgesic ladder for cancer pain management published in 1986 [62]. The WHO guidance has been updated recently and, while still emphasising a stepwise approach, the guidelines prioritise individualised, comprehensive pain management, balancing analgesic efficacy with an aim to minimise adverse effects [63].

Opioids are effective in treating moderate to severe acute pain and in the oncological patient population they remain a mainstay of analgesia. There has been specific interest in the direct and indirect impact of opioids on both primary tumour development and metastatic potential. There is abundant evidence of wide‐ranging influences in vitro and in vivo studies on the immunomodulatory effects of opioids, although there is a degree of inter‐drug variability [64]. Impairment of the innate and adaptive immune systems occurs through a number of mechanisms including reductions in gut barrier mucus production; changes in natural killer cell and macrophage function; and the ability of cells to present antigens [62, 63, 64, 65, 66, 67]. However, in the context of acute pain (which is also immunosuppressive), opioid administration in analgesic doses may be immunoprotective [68], thus arbitrating on the relative risks and benefits of opioid use in patients with cancer. The clinical relevance of these findings remains opaque, with large population‐level studies failing to show an increased risk of breast cancer recurrence in patients taking opioids [69] or increased risks of malignancy in those patients taking opioids for persistent pain [70]. Where evidence remains contradictory, a strategy using the lowest possible dose, initially in an immediate‐release preparation, is supported by recent guidelines [71].

Neuropathic features of acute cancer pain occur in approximately 40% of patients with cancer [72]. Mechanisms are complex, potentially involving direct tumour involvement; nerve compression or infiltration; chemotherapy and/or radiotherapy‐induced nerve damage; or post‐surgical complications [73]. Neuropathic cancer pain exists commonly in conjunction with nociceptive pain and therefore responds better to combination therapy.

The evidence for preventative and therapeutic strategies is limited and utilises management strategies for similar neuropathic states. Tricyclic antidepressants and serotonin noradrenaline reuptake inhibitors act at a spinal and supraspinal level by enhancing the effect of the descending inhibitory pathways due to an increase in noradrenaline and serotonin, showing analgesic effects independent of their antidepressant effect. Where there is intolerance to tricyclic antidepressants, serotonin noradrenaline reuptake inhibitors may be a useful alternative. Duloxetine and venlafaxine have shown some effectiveness in chemotherapy‐induced neuropathic pain. An alternative option to antidepressants are the gabapentinoids (pregabalin, gabapentin and to a lesser extent mirogabalin) [74]. These drugs act by modulating spinal pain pathways as well as stimulating descending inhibitory pathways thereby reducing neuronal hyperactivity – a major pathophysiological mechanism of neuropathic cancer pain. There has been interest in the opioid‐sparing effects of the gabapentinoids in acute postoperative pain but evidence supporting their systematic use in such a role is minimal [75]. Ketamine, a phencyclidine derivative, is used clinically as an analgesic drug for acute and chronic pain [76]. It acts as a selective antagonist of the ionotropic N‐methyl‐_D_‐aspartate (NMDA) glutamate receptor resulting in hyperpolarisation of neuronal cell membranes [77]. It has shown particular utility when combined with opioids for the management of mucositis in children [78].

Aside from pharmacotherapy, interventional techniques can provide benefit for acute oncological pain. The most satisfactory outcomes are achieved after thorough assessment and careful patient selection. Considerations at assessment include previous treatments; the type and mechanism of pain; patient factors and preference; expected survival; availability of skills; and the resources to safely perform the chosen technique. Risks vs. potential benefits need to be considered on a case‐by‐case basis since there are complications associated with interventions. Some of the more commonly performed acute cancer pain procedures are outlined below.

Neuraxial techniques include intrathecal and epidural routes. They are usually considered in patients who have escalating opioid requirements and who show little response or poor tolerance. Significantly lower doses of medication are required when deployed neuraxially when compared with systemic routes of administration. For patients with a significant acute oncological pain flare up, a tunnelled epidural or intrathecal catheter connected to an external pump may provide a vital respite from severe and debilitating pain [79]. Patients with longer expected survival rates (> 3 months) may potentially benefit from an implantable device such as an intrathecal drug delivery system [80]. In the majority of cases, opioids (morphine, hydromorphone) as well as a local anaesthetic are infused and considered first‐line. However, adjuvants such as ziconotide (presynaptic calcium channel blocker), clonidine and ketamine can be considered to reduce opioid requirements [81].

Peripheral blocks applied to cancer‐related pain include paravertebral; erector spinae plane; brachial plexus; nerves in the head and neck; and intercostal nerves. The evidence for their use in acute cancer‐related pain is, however, limited to single cases and smaller series which illustrate some benefit [82]. Local anaesthetics are used most commonly for these blocks but due to their short duration of action may be given as intermittent boluses or continuously using a catheter, with or without adjuvants such as adrenaline and clonidine. Neurolytic drugs have also been used in some documented cases but are rarely implemented in those patients with a good prognosis as resulting neuritis can cause more problematic symptoms than the original pain.

Sympathetic nerve blocks are useful as visceral afferent nociception is mediated by the sympathetic nervous system which can be readily interrupted, providing analgesia. Coeliac plexus and splanchnic neurolytic blocks with ethanol/alcohol may be considered for upper abdominal cancers such as pancreas, stomach and liver, especially in those patients with short life expectancy. However, results have been mixed especially where tumours are more advanced, with distortion of local surrounding anatomy leading to variable spread of neurolytic drugs. For lower abdominal and pelvic structures such as the bladder, prostate, sigmoid colon and rectum, the afferent nociceptive signals pass through the superior hypogastric plexus which can be blocked. Ganglion of impar blocks are most suitable for perineal structures. Sympathetic nerve blocks are performed routinely under imaging and contrast guidance, traditionally X‐ray, but now many are performed under computed tomography guidance, leading to improved accuracy.

Minimally invasive procedures for vertebral pain arising from metastatic collapse include vertebroplasty (percutaneous injection of cement into vertebral bodies); kyphoplasty (balloon inflation into vertebral body to create space for cement to correct vertebral height) [83]; radiofrequency ablation (using heat generated from alternating current) [84]; and cryoablation (use of cryoprobes with cooled conductive fluid) [85] for cancer‐related bone pain caused by pathological compression fractures or vertebral metastases. Careful patient selection is important to ensure benefit is gained from these procedures.

Research and clinical applications of psychological approaches to pain management have focused predominantly on chronic pain, where cognitive behavioural therapy and third‐wave cognitive behavioural therapies (e.g. acceptance and commitment therapy and mindfulness‐based approaches) have been implemented [86, 87, 88]. These form the basis of psychological acute pain management, with their application usually occurring in a more limited timeframe within the inpatient environment [89]. It is helpful for the psychological component of the assessment (which may include collateral information from hospital staff, relatives or friends) to identify: the patient's pain perceptions (e.g. cause, identity, symptoms, control and timeline); current and historical mental health difficulties and treatments, such as depression (where pessimism, hopelessness and low motivation can reduce adherence to treatment), anxiety or trauma (for awareness of any tendency to catastrophise or to be hypervigilant to bodily sensations and other threats); behavioural changes and coping strategies in response to the pain; and substance use (to manage the potential risks of self‐medication, over‐ or under‐use of analgesia). Following assessment, it is important to share with the patient the provisional biopsychosocial formulation of the acute pain and to update this based on patient feedback to ensure a collaboratively developed understanding of the acute pain and its management. A diagrammatic/pictorial formulation can be more accessible to patients acting later as an aide‐memoire. This collaborative and transparent stance enhances the patient's relationship with healthcare staff alongside their motivation and adherence to healthcare guidance. It will also present the rationale for any intervention, including onward referral to specialist services for pre‐existing difficulties.

Patient education is key to psychological acute pain management. Expanding, correcting or reinforcing the patient's understanding of the cause of the pain, its symptoms, likely duration and what they and healthcare staff can do to manage it, can provide a framework for them to make sense of their experience. Information on the ‘fight or flight’ response can help to understand the link between physiology, thoughts, emotions and behaviour; this can reduce potential misinterpretations of the pain experience which may otherwise increase distress [90] and can clarify why thoughts and emotions can exacerbate and ameliorate pain. Understanding the function of pain and what they should do (or not do) behaviourally in the short‐, medium‐ and longer‐term can help to reduce fear‐avoidant behaviour. Patients may need to acquire or enhance existing skills, such as self‐compassion, to increase awareness of their thoughts and feelings to enable them to identify catastrophic misinterpretations and/or to identify thoughts that may be accurate but unhelpful at the current time. They may also need to implement relaxation techniques (e.g. diaphragmatic breathing and imaginal relaxation). All of these skills can disrupt the ‘fight or flight’ response and enhance a patient's sense of control, which in turn can reduce distress and facilitate engagement with healthcare staff and supportive family and friends.

Limits to psychological approaches include the patient wanting, or being able, to engage with it, which may not be possible when an individual is in too much pain or in the later stages of cancer. Nonetheless, a recent service evaluation of psychological approaches to acute (non‐cancer) pain management found cost effective reductions in hospital admissions and bed days. However, more work is required to ascertain the efficacy of psychological approaches to manage acute pain.

## Conclusions

**Figure 1:**
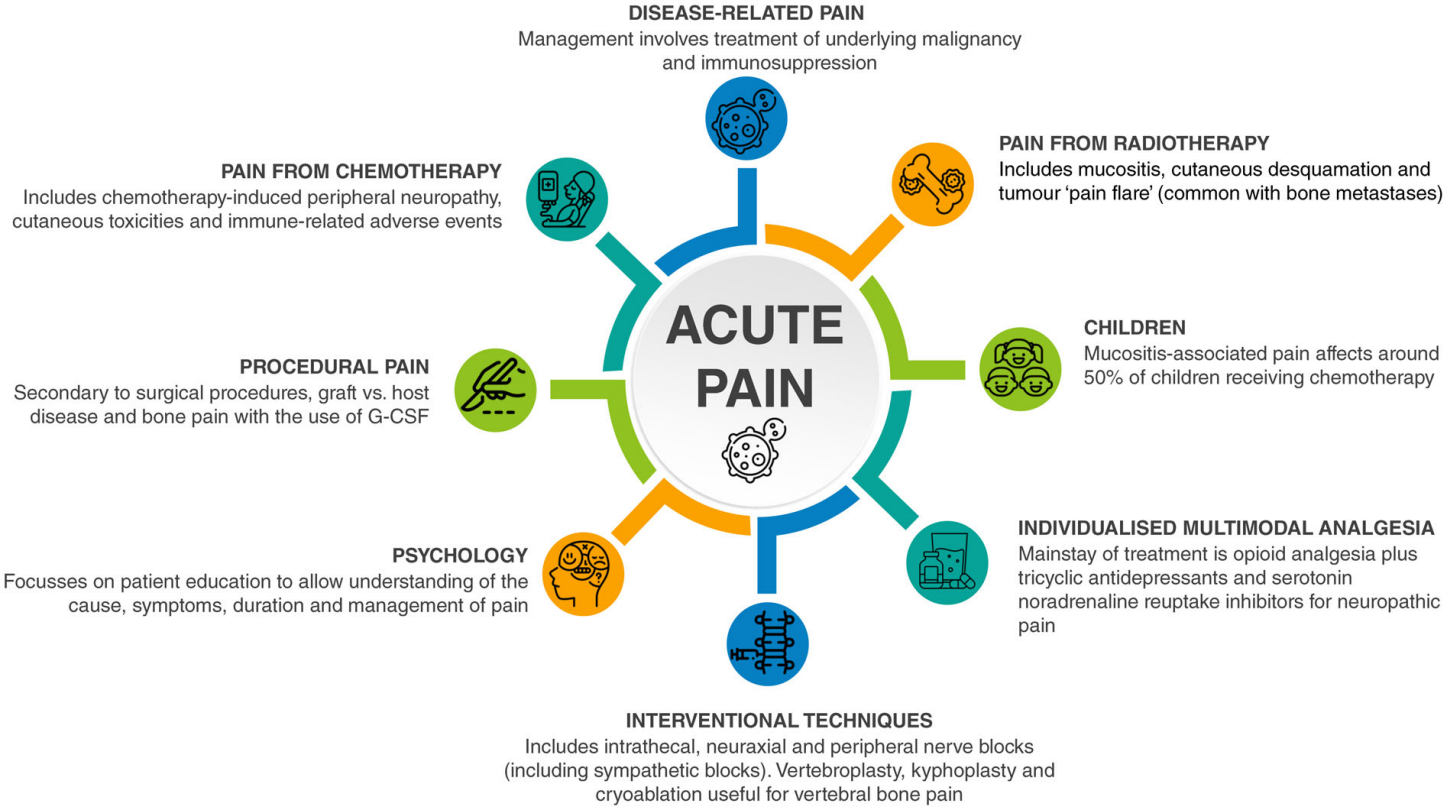
Summary infographic of the causes and management of acute oncological pain. G‐CSF, granulocyte colony stimulating factor.

Myriad acute pain causes in the oncology population can occur concurrently or sequentially and are often interrelated. While the underlying causes of the acute pain may differ, the pain itself often has neuropathic features and can prove challenging to treat. These acute pain states occur commonly in, or present to, healthcare settings and teams with limited experience in managing complex pain. This is a situation which may lead to acute pain in oncology patients being controlled suboptimally. Understanding the causes and taking a biopsychosocial management approach are key to effective analgesia. The main findings of our review are summarised in Fig. 1.

Acute pain in the oncology patient is often perceived to represent the mere foothills of a more challenging mountain range (that of persistent pain) encountered in those patients who are considered to be ‘cancer survivors’, and therefore have completed all or the majority of their oncological management. Given the fact that poorly controlled acute pain may have a deleterious impact on individual patients' oncology outcomes, we believe that its optimal control should form a key component of a patient's oncological treatment.
